# Predictive Efficacy of Blood-Based Tumor Mutation Burden Assay for Immune Checkpoint Inhibitors Therapy in Non-Small Cell Lung Cancer: A Systematic Review and Meta-Analysis

**DOI:** 10.3389/fonc.2022.795933

**Published:** 2022-02-09

**Authors:** Nan Zhang, Jinwei Zhang, Guoqing Wang, Xin He, Yin Mi, Ying Cao, Xiaoxu Yu

**Affiliations:** ^1^ Department of Clinical Laboratory, First Teaching Hospital of Tianjin University of Traditional Chinese Medicine, National Clinical Research Center for Chinese Medicine Acupuncture and Moxibustion, Tianjin, China; ^2^ Department of Cardiothoracic Surgery, Tianjin Medical University General Hospital, Tianjin, China; ^3^ Tianjin Key Laboratory of Oral and Maxillofacial Function Reconstruction, Tianjin Stomatological Hospital, Hospital of Stomatology, Nankai University, Tianjin, China; ^4^ Department of Radiotherapy, First Affiliated Hospital of Zhengzhou University, Zhengzhou, China; ^5^ Department of Clinical Laboratory, Tianjin Central Hospital of Gynecology Obstetrics, Tianjin, China

**Keywords:** tumor mutation burden (TMB), liquid biopsy, immune checkpoint inhibitors (ICIs), non-small cell lung cancer (NSCLC), meta-analysis

## Abstract

**Background:**

In non-small cell lung cancer (NSCLC) patients treated by immune checkpoint inhibitors (ICIs), tumor mutation burden (TMB) has been found to have predictive potential for survival. When compared to TMB detection in tissue (tTMB), detecting TMB in the blood (bTMB) has practical advantages; yet, the results of various studies are conflicting. The question of whether bTMB can be utilized as a predictive biomarker is becoming increasingly contentious. To confirm the predictive efficacy of bTMB, researchers did a systematic review and meta-analysis to look into the relationship between ICIs and bTMB.

**Method:**

From the inception to March 2021, Cochrane Library, PubMed, EMBASE and other databases were systematically searched. The predictive value of bTMB in ICIs, or the efficacy of ICIs against chemotherapy, was studied. The results were presented as pooled ratio rate (RR) and hazard ratio (HR) with 95% confidence intervals for the Objective response rate (ORR), progression-free survival (PFS), and overall survival (OS). Subgroup analysis, heterogeneity analyses, and sensitivity analysis were also performed.

**Results:**

A total of 2,610 NSCLC patients were studied in seven trials. There were no significant differences in OS (HR = 1.09; 95% CI: 0.62–1.91, *P* = 0.774) or PFS (HR = 0.73; 95% CI: 0.20–2.65, *P* = 0.629) between high and low bTMB groups in the ICIs cohort. When ICIs were compared to chemotherapy, ICIs were found to enhance OS (HR = 0.74; 95% CI: 0.59–0.92, *P* = 0.006), but the improvement in PFS and ORR was only a numerical trend (PFS: HR = 0.83; 95% CI: 0.63–1.09, *P* = 0.173; ORR: RR = 0.92, 95% CI: 0.77–1.10, *P* = 0.372). NSCLC patients treated with ICIs in the high bTMB group had better survival benefits than chemotherapy patients in terms of OS (HR = 0.63; 95% CI: 0.51–0.76, *P <*0.001), PFS (HR = 0.63; 95% CI: 0.52–0.76, *P <*0.001), and ORR (RR = 1.86; 95% CI: 1.32–2.62, *P <*0.001), while in the low TMB group, the results were no different or even reversed (OS: HR = 0.89; 95% CI: 0.64–1.24, *P* = 0.485; PFS: HR = 1.21, 95% CI: 0.93–1.58, *P* = 0.154; ORR: RR = 0.68, 95% CI: 0.54–0.85, *P* = 0.001).

**Conclusions:**

TMB could predict the enhanced survival benefit of NSCLC patients treated with ICIs; however the role of bTMB is limited at this stage. For NSCLC patients with high TMB, ICIc may be a better option than chemotherapy.

## Introduction

Immunotherapy has attracted increasing attention from clinical oncologists in recent years as a revolutionary therapy, particularly with immune checkpoint inhibitors as a successful representative. Immune checkpoint inhibitors (ICIs) that target programmed cell death-1 (PD-1) or its ligand (PD-L1) as monotherapy or in combination with anticytotoxic T-lymphocyte-associated antigen-4 therapy have been shown to be more effective than standard therapies in several cancers, particularly non-small cell lung cancer (NSCLC) (1–4). However, a large proportion of patients do not respond to immunotherapy, and response rates in most cancer types are only around 20% in unselected patient populations (5). Although combination immunotherapy can improve response rates, it also increases the occurrence of immune-related adverse events (irAEs) (6, 7). Furthermore, the cost of these therapies is so high (close to $150,000 per patient per year) that it frequently places a severe financial burden on patients (8). As a result, identifying predictive biomarkers to identify responsive patients is critical.

Several biomarkers, including but not limited to the status of microsatellite instability (MSI)/DNA mismatch repair (MMR) and PD-L1 expression, have been reported to predict ICI efficacy in multiple malignancies. Currently, the Food and Drug Administration (FDA) has approved the two biomarkers as predictive biomarkers for immunotherapy in certain cancers (9–11). The clinical application of these biomarkers, however, is difficult due to their inherent limitations. MSI-high (MSI-H)/MMR deficiency (dMMR) is a rare occurrence (0.6%) in NSCLC, so the application in clinical testing is not common. Patients who were PD-L1 positive had a 15–31% chance of benefiting from ICIs, despite the fact that PD-L1 expression is a widely accepted biomarker for predicting ICI benefit in NSCLC. However, 10–24% of patients with negative expression responded to ICI monotherapy (12–14). Because of the presence of various flaws, the predictive value of these two biomarkers has been questioned. Tumor mutation burden (TMB), a surrogate for tumor neoantigen load derived from the total somatic mutation count, has been shown in clinical trials to correlate with ICI efficacy (15, 16). Recent research has found that higher TMB predicts better clinical benefit in melanoma (17), NSCLC (18–20), urothelial carcinoma (21) and other cancers. There is currently no unified standard for TMB detection. Previous studies used whole exome sequencing (WES) or targeted panel-based sequencing from tumor tissue samples to determine TMB. However, it is unfortunate that obtaining adequate tissue samples is frequently clinically impossible, particularly for patients with advanced or metastatic cancers (22, 23). Furthermore, because of their invasiveness and risk of complications, biopsy techniques pose logistical and ethical challenges (24). Furthermore, biopsy specimens that have been archived do not always correspond to the molecular features of the disease at the time of treatment initiation. TMB measurement from blood specimens (bTMB) has also emerged in recent years (25, 26). Although blood-based TMB shows promising results, there is still debate about whether bTMB assessed by panel-based targeted sequencing has potential predictive power in NSCLC patients treated with ICIs (27, 28). As a result, it is becoming increasingly urgent and important to investigate the true role of bTMB in ICI-treated NSCLC patients.

In summary, we conducted a systematic review and meta-analysis to assess the value of bTMB in order to better understand the predictive efficacy of bTMB on ICI therapy in patients with NSCLC.

## Materials and Methods

### Literature Search and Study Selection

A comprehensive systematic review and meta-analysis were carried out in accordance with the Preferred Reporting Items for Systematic Reviews and Meta-Analyses (PRISMA) guideline (29). PubMed, EMBASE, Cochrane Library, SinoMed, and CNKI were systematically searched to identify relevant studies published between inception and March 2021, with no language restrictions. The databases were searched independently by two investigators (NZ and XY). The following were the key search terms: “tumor mutation burden” OR “tumor mutational burden” OR “tumor mutation load” OR “tumor mutational load” OR “TMB” OR “TML”. Initially, articles were screened using the title and abstract; then, eligible articles were evaluated using the full text. We also looked through the reference lists of the included articles to find any missing literature.

To identify eligible studies, the following inclusion criteria were used: 1) patients with NSCLC who accepted ICIs treatment; 2) TMB is precisely defined, and the sample used to test TMB must be blood; 3) at last one or more main evaluation indicators [progression-free survival (PFS), overall survival (OS) or Objective response rate (ORR)]were available comparing low bTMB against high bTMB in ICIs cohort group or comparing ICIs treatment and chemotherapy (any kind of chemotherapy); 4) the hazard ratio (HR) with 95 percent confidence interval (CI) of OS and PFS, as well as the relative risk (RR) of ORR, could be obtained directly; and 5) only recent studies were chosen when authors from the same institution published multiple articles.

Due to a lack of information, reviews, guidelines, letters, expert opinion, comments, meeting abstracts, animal studies and so on were excluded.

### Data Extraction and Assessment of the Quality of the Included Studies

Two investigators (NZ and XY) independently reviewed the included studies and extracted the following data: the surname of the first author, publication year, type of study, sample size, study design, country of origin, drugs, line of therapy, bTMB cutoff value, and the main reporting outcomes. HR and 95% CI for OS and/or PFS or RR for ORR were extracted for pooled analysis. The two investigators (NZ and XY) then independently examined all of the data. Any disagreements were discussed with another author (XH) and resolved. The Newcastle–Ottawa Scale (NOS) recommended by the Cochrane Non-Randomized Studies Methods Working Group (30) and Cochrane Collaboration’s Tools (31) were used to assess the methodology quality of non-randomized trials and randomized controlled trials for meta-analysis. The NOS is made up of three quality parameters: selection (0–4 points), comparability (0–2 points), and outcome assessment (0–3 points). The total NOS scores ranged from 0 to 9, with higher scores indicating higher quality. Methodological studies with a score of 6 are of high quality. The randomized controlled studies were evaluated using the Cochrane Collaboration’s Tools, which included seven items such as random sequence generation, allocation concealment, blinding of participants and personnel, blinding of outcome assessment, incomplete outcome data, selective reporting, and other bias. Each item was categorized as low, high, or unclear risk of bias.

### Statistical Evaluation

The combined HRs and 95% CIs extracted from each eligible study were used to assess the relationship between TMB and OS/PFS. To estimate the relationship between TMB and ORR, the combined RRs and their 95% CI were combined. Forest plots were used to estimate the overall HR/OR and its 95% CI. The combined HR<1 with a 95% CI less than one or the combined RR>1 with a 95% CI greater than one indicated an improved survival benefit for patients with high TMB. A *Z*-test was used to determine the statistical significance of the pooled HR and RR. If *P <*0.05, the results are considered statistically significant. The *Q* test and *I^2^* value, which is a quantitative measure of inconsistency across studies, were used to assess study heterogeneity. The fixed effect model (Mantel–Haenszel method) was used if *P <*0.10 in the *Q* test or *I^2^* was <50% (32).. Otherwise, a random effect model analysis was carried out. Sensitivity analyses were used to investigate the source of heterogeneity. STATA software (version 14.0; Stata Corporation, College Station, TX) was used for all statistical analyses and *P <*0.05 was considered statistically significant.

## Results

### Literature Search and Study Characteristics

**Figure 1:**
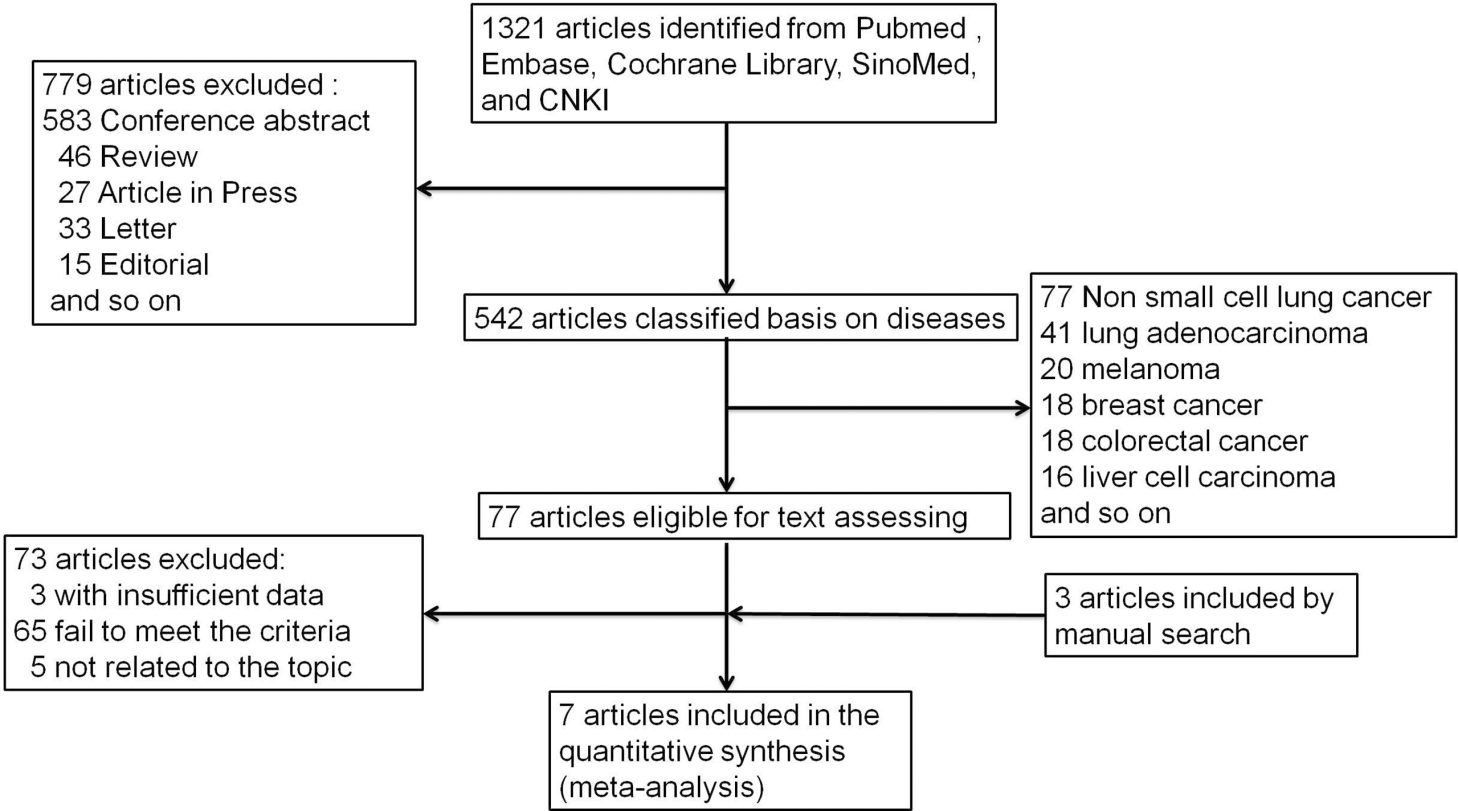
Flow chart of literature search.

**Figure 1** depicts the process of conducting a literature search. In the initial electronic search of the major database, 1,321 papers were found. After excluding reviews, letters, conference abstracts, and other items, 779 papers were left out. The remaining 542 publications were classified according to disease species. There were 77 papers related to non-small cell lung cancer among them. Following that, we carefully reviewed the remaining 77 publications, and 73 were eliminated due to failing to meet the criteria. According to the references, there were three publications that could meet the demand. The meta-analysis eventually included seven articles (25, 27, 28, 33–36) published between 2018 and 2020. **Table 1** lists the main characteristics of the included studies. Four of the seven included studies were retrospective, two were randomized controlled trials, and one was a prospective study involving 2,610 NSCLC patients. Three multicenter studies were conducted: two in China and two in the United States. Patients in two studies were at stage IV of the tumor, patients in four studies were at stages III–IV, two studies did not specify a stage, and patients in one study were at stages I–IV. In all of the literatures, bTMB detection is based on the next-generation sequencing (NGS) gene panel assays. The definitions of high and low bTMB varied across studies. The cutoff value for bTMB ranged from 6 to 20. Wang (27) and Gandara (25) used different research objects in the same study, out of all the studies included.

**Table 1:** The main feature of the studies included in the meta-analysis.

Study	Year	Type of study	N	Region	Tumor stage	Experiment drugs	Line of therapy	bTMB Cutoff value	Outcome
Herbst (33)	2020	Randomized	389	Multiple areas	IV	Atezolizumab	1st	16	PFS, OS
Rizvi (34)	2020	Randomized	809	Multiple areas	IV	Durvalumab or Durvalumab plus Tremelimumab	1st	20	PFS, OS, ORR
Wang (27)	2020	Retrospective	64	China	III–IV	ICIs	All	6	PFS, OS, ORR
Wang_(POPLAR and OAK)_ (27)	2020	Retrospective	429	Multiple areas	III–IV	Atezolizumab	2st	16	PFS, OS, ORR
Aggarwal (35)	2020	Prospective	26	USA	NA	Pembrolizumab	1st	16	PFS, OS
Fang (36)	2020	Retrospective	72	China	III–IV	ICIs	NA	13	PFS
Chae (28)	2019	Retrospective	27	USA	I–IV	ICIs	All	14.5	PFS, OS
Gandara (25)	2018	Retrospective	211	Multiple areas	NA	Atezolizumab	2st	16	PFS, OS
Gandara (25)	2018	Retrospective	583	Multiple areas	III–IV	Atezolizumab	2st	16	PFS, OS, ORR

NA, unavailable.

### Evaluation of Quality

All seven studies were evaluated using the Cochrane Collaboration’s tools and the NOS. The results in **Tables 2**, **3** show that all of the included studies were of moderate to high quality.

**Table 2:** The Newcastle–Ottawa Scale (NOS) assessment of the included studies’ risk of bias.

Study	Selection	Comparability	Outcome	Total score*
Aggarwal	3	1	3	7
Chae	3	1	3	7
Fang	3	1	3	7
Gandara	3	1	2	6
Wang	4	1	3	8
Wang(POPLAR and OAK)	3	1	2	6

*NOS points: 0 to 3: very high risk of bias; 4 to 6: high risk of bias; 7 to 9: low risk of bias.

**Table 3:** The Cochrane collaboration’s model for assessing risk of bias of randomized controlled trials.

Study	Random sequence generation	Allocation concealment	Blinding of participants and personnel	Blinding of outcome assessment	Incomplete outcome data	Selective reporting	Other bias
Herbst	Low risk	Low risk	High risk	Low risk	Low risk	Low risk	Low risk
Rizvi	Low risk	Low risk	High risk	Low risk	Low risk	Low risk	Low risk

#### Blood-Based TMB Predictive Efficacy of ICI Treatment in NSCLC Patients

**Figure 2:**
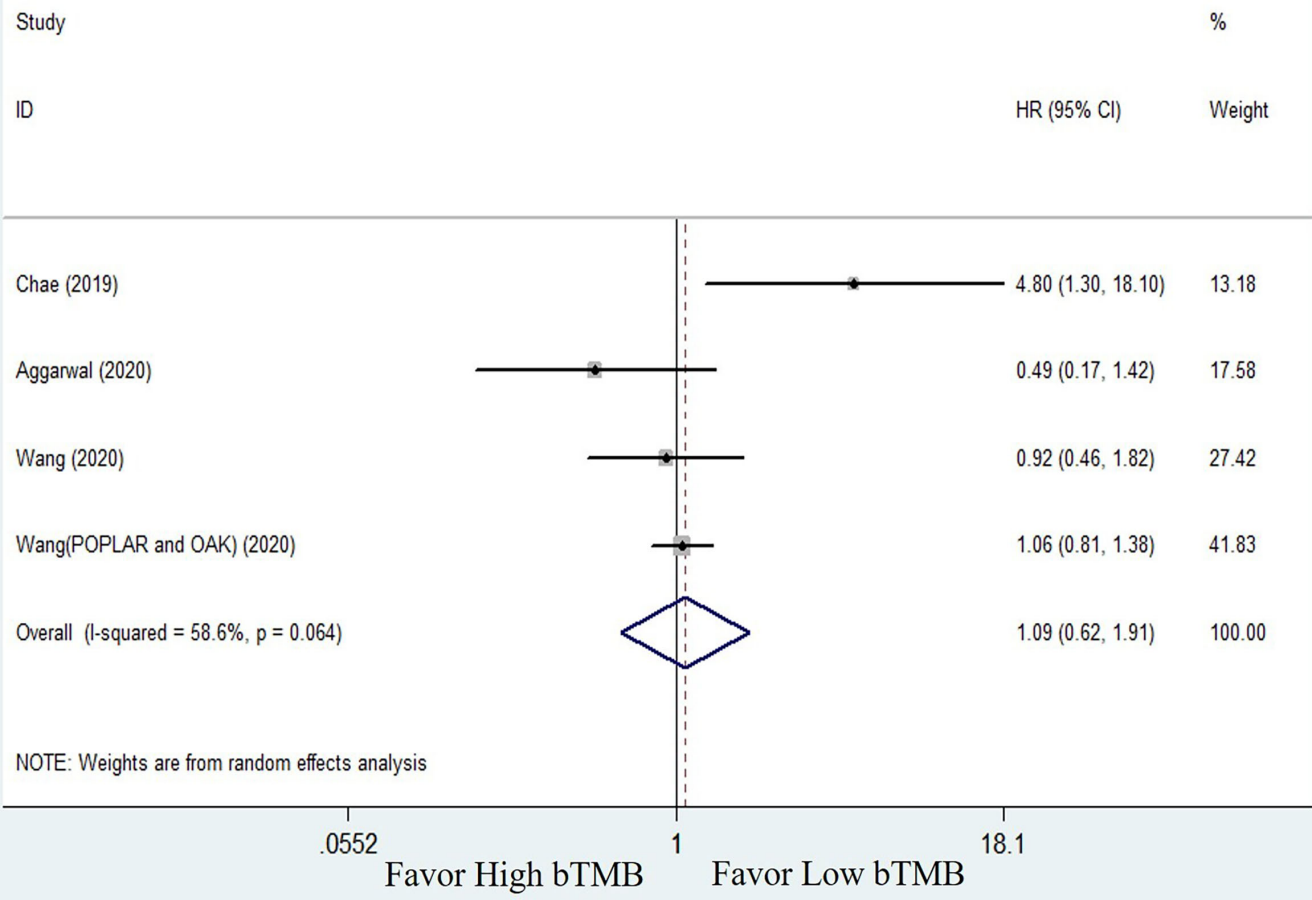
Forest plot of clinical benefits’ comparison for overall survival (OS) between high bTMB group and low bTMB group.

**Figure 3:**
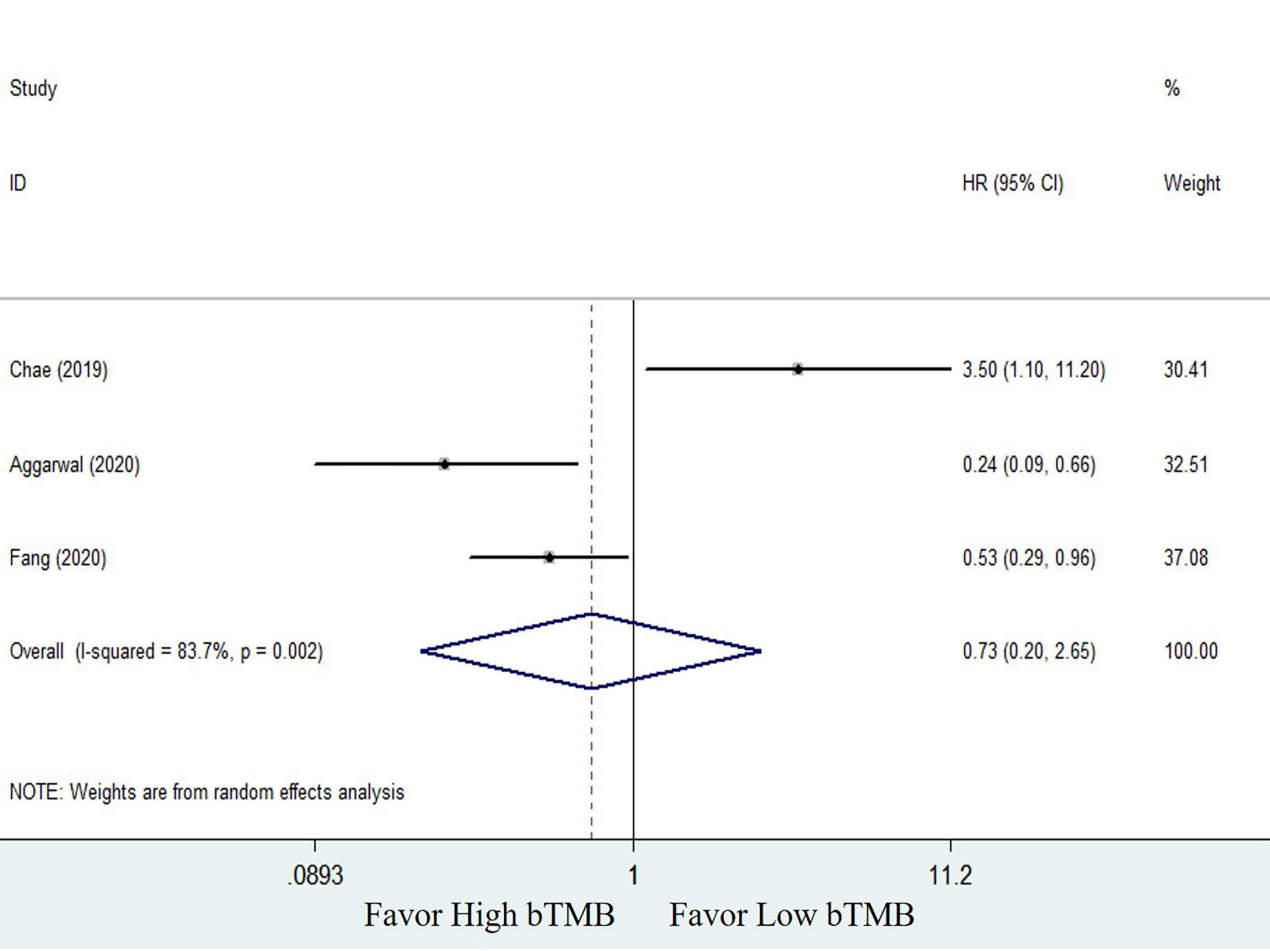
Forest plot of clinical benefits’ comparison for progression-free survival (PFS) between high bTMB group and low bTMB group.

The clinical benefits of NSCLC patients with different TMB levels were evaluated to validate the feasibility of bTMB as a predictor. Clinical benefit indicators such as overall survival (OS) and progression-free survival (PFS) were compared between low TMB and high TMB patients. Only three of the seven studies involved in the bTMB assay (27, 28, 35) with four sets of data totaling 546 patients provided enough information to evaluate the relationship between OS and TMB. As shown in **Figure 2**, meta-analysis revealed no significant difference between NSCLC patients with low bTMB and those with high bTMB. The pooled HR was 1.09 [95% confidence interval (CI) 0.62–1.91; *P* = 0.774]. Because there was significant heterogeneity between the studies (*I^2^* = 58.6%, *P* = 0.064), a random-effects model was used. Three studies (28, 35, 36) with a total of 125 patients were conducted to assess the relationship between PFS and bTMB. The heterogeneity was significant, so a random-effects model was used (*I^2^* = 83.7%, *P* = 0.002). The pooled HR was 0.73 [95% CI 0.20–2.65; *P* = 0.629] as seen in the forest plot (**Figure 3**). Similarly, no statistically significant differences were found between low and high bTMB NSCLC patients. As a result, blood-based TMB did not appear to be effective in screening NSCLC patients for ICIs.

#### Comparison of Efficacy of ICIs and Chemotherapy and Subgroup Analysis Based on bTMB Levels

**Figure 4:**
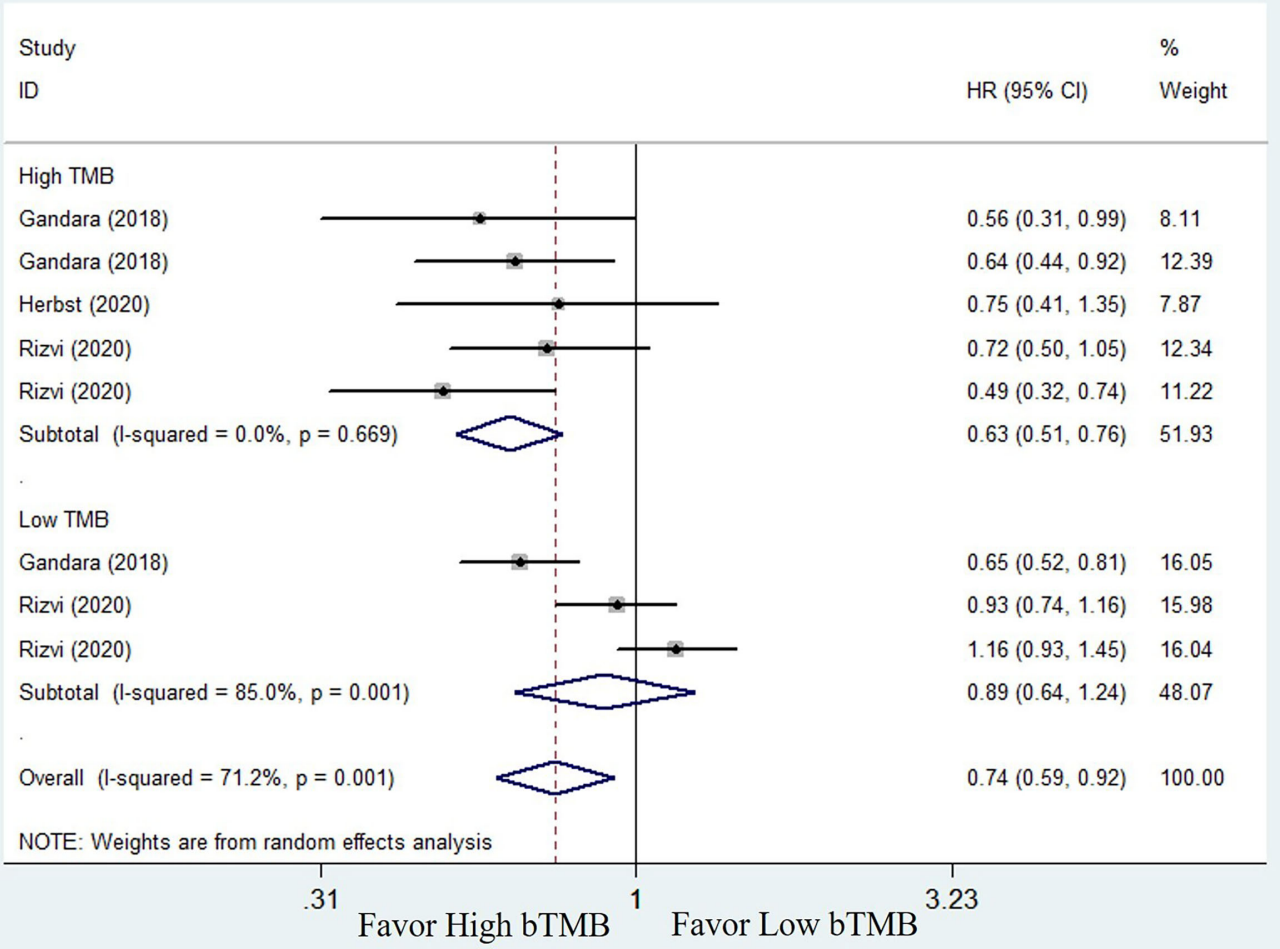
Forest plot of efficacy comparison of ICIs to chemotherapy for overall survival (OS) and subgroup analysis.

**Figure 5:**
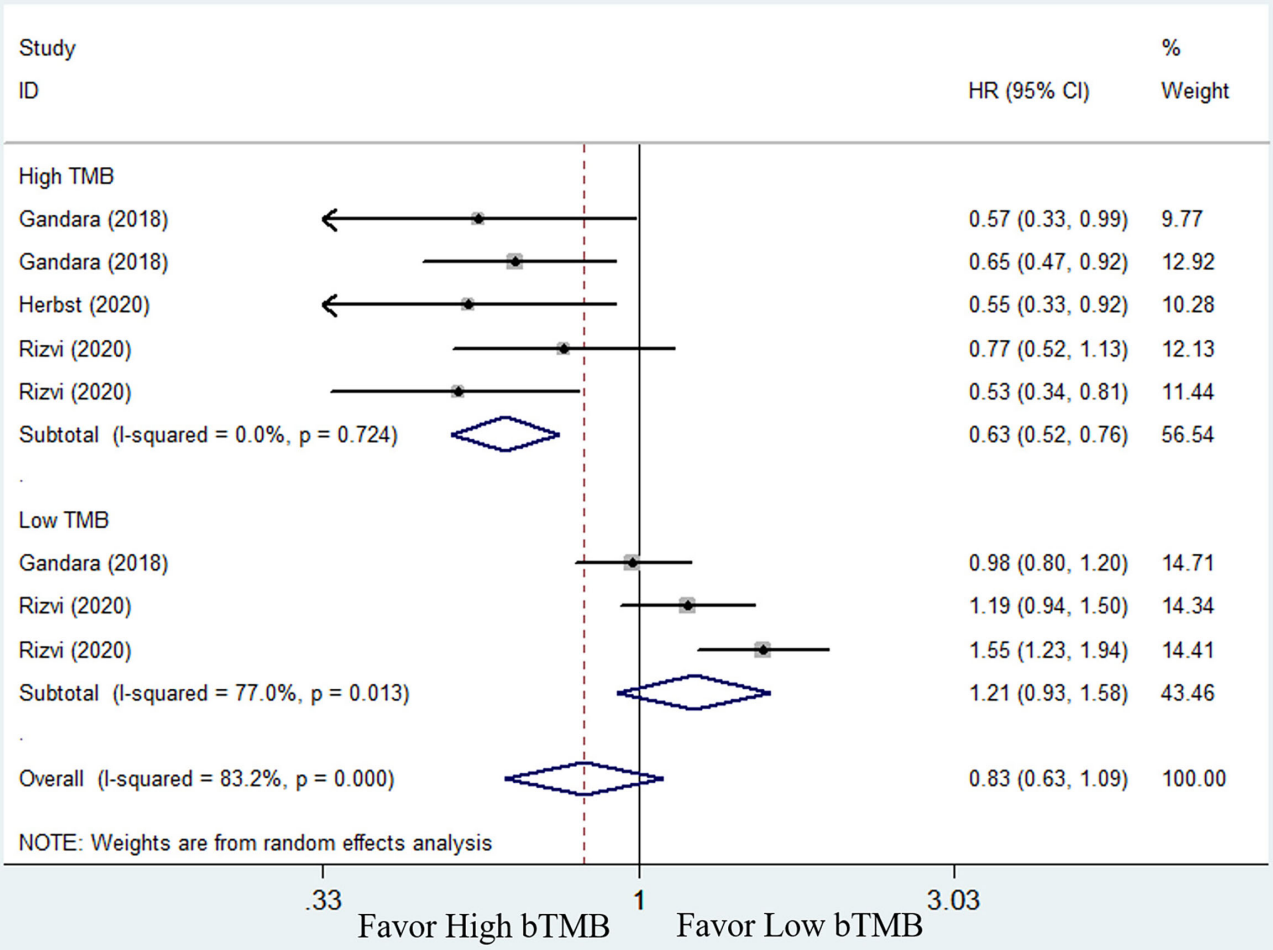
Forest plot of efficacy comparison of ICIs to chemotherapy for progression-free survival (PFS) and subgroup analysis.

**Figure 6:**
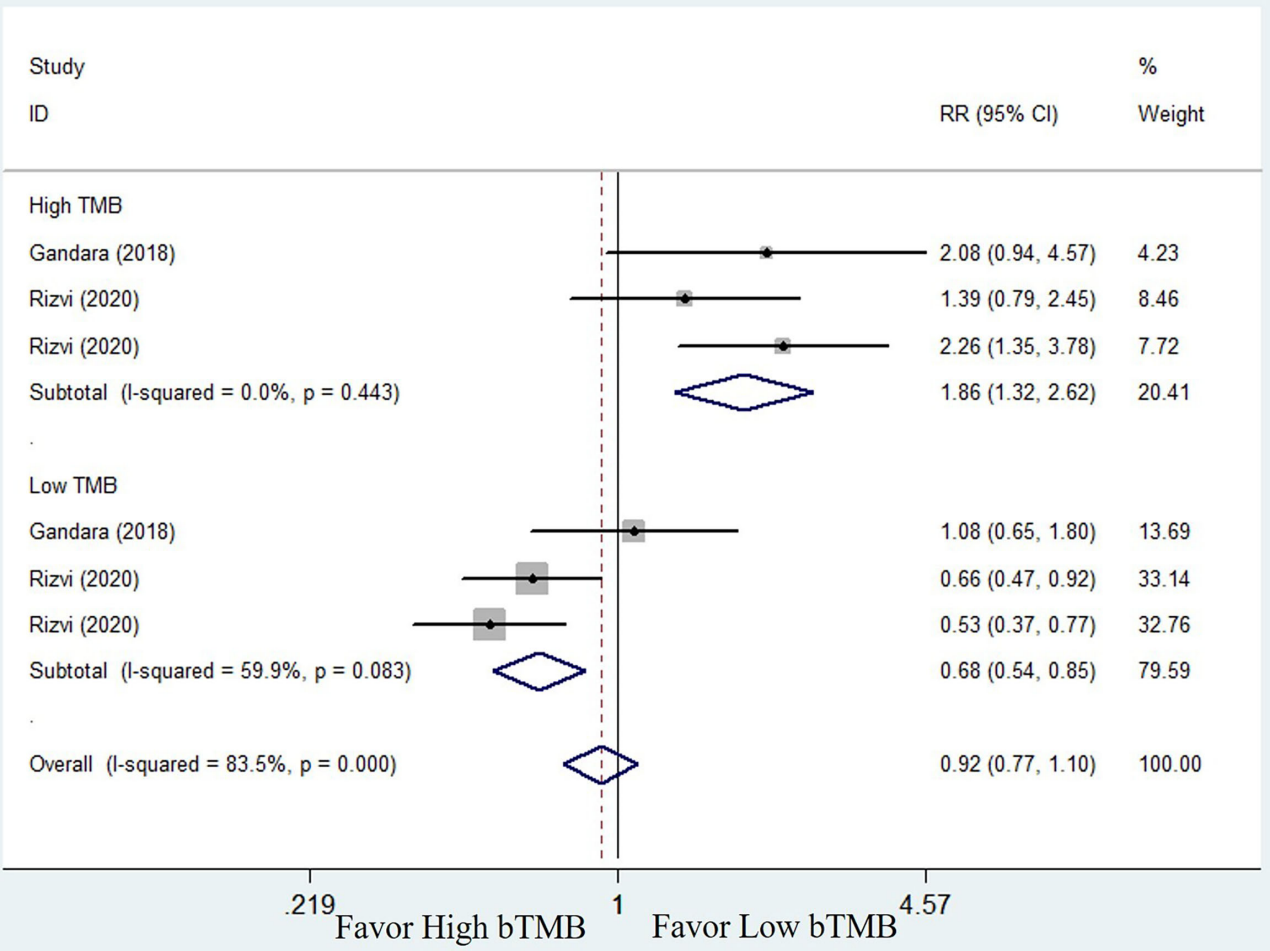
Forest plot of efficacy comparison of ICIs to chemotherapy for objective response rate (ORR) and subgroup analysis.

To compare the efficacy of chemotherapy and ICIs based on bTMB as a biomarker in NSCLC patients, we performed a pooled and subgroup analysis based on bTMB levels. There were three trails (25, 33, and 34) with multiple sets of data to evaluate the PFS, ORR, and OS. The pooled outcome for OS suggested that ICIs therapy can significantly improve long-term survival status of NSCLC patients when compared to chemotherapy (HR = 0.74, 95% CI: 0.59–0.92, *P* = 0.006; *I^2^* = 71.2%, random-effects). Subgroup analysis revealed that patients with high bTMB improved more than patients with low bTMB (high bTMB: HR = 0.63, 95% CI: 0.51–0.76, *P <*0.001; low TMB: HR = 0.89, 95% CI: 0.64–1.24, *P* = 0.485) (**Figure 4**). PFS, on the other hand, demonstrated only a numerical improvement, not a statistical difference. The pooled HR was 0.83 (95% CI: 0.63–1.09, *P* = 0.173), with significant heterogeneity (*I^2^* = 83.2%, random-effects). However, subgroup analysis revealed that patients with high bTMB could benefit more from ICIs than chemotherapy (HR = 0.63; 95% CI: 0.52–0.76, *P <*0.001), but not patients with low bTMB (HR = 1.21, 95% CI: 0.93–1.58, *P* = 0.154) (**Figure 5**). A similar situation occurred in the ORR. Although there was no overall statistical difference between ICIs and chemotherapy (RR = 0.92, 95% CI: 0.77–1.10, *P* = 0.372), patients with high bTMB responded better to ICIs than chemotherapy (RR = 1.86, 95% CI: 1.32–2.62, *P <*0.001). Patients with low bTMB, on the other hand, responded better to chemotherapy than ICIs, and the difference was significant (RR = 0.68, 95% CI: 0.54–0.85, *P* = 0.001) (**Figure 6**). To summarize, ICI treatment improves overall survival benefit in NSCLC patients with high bTMB compared to chemotherapy, but not in patients with low bTMB.

### Sensitivity Analysis

**Figure 7:**
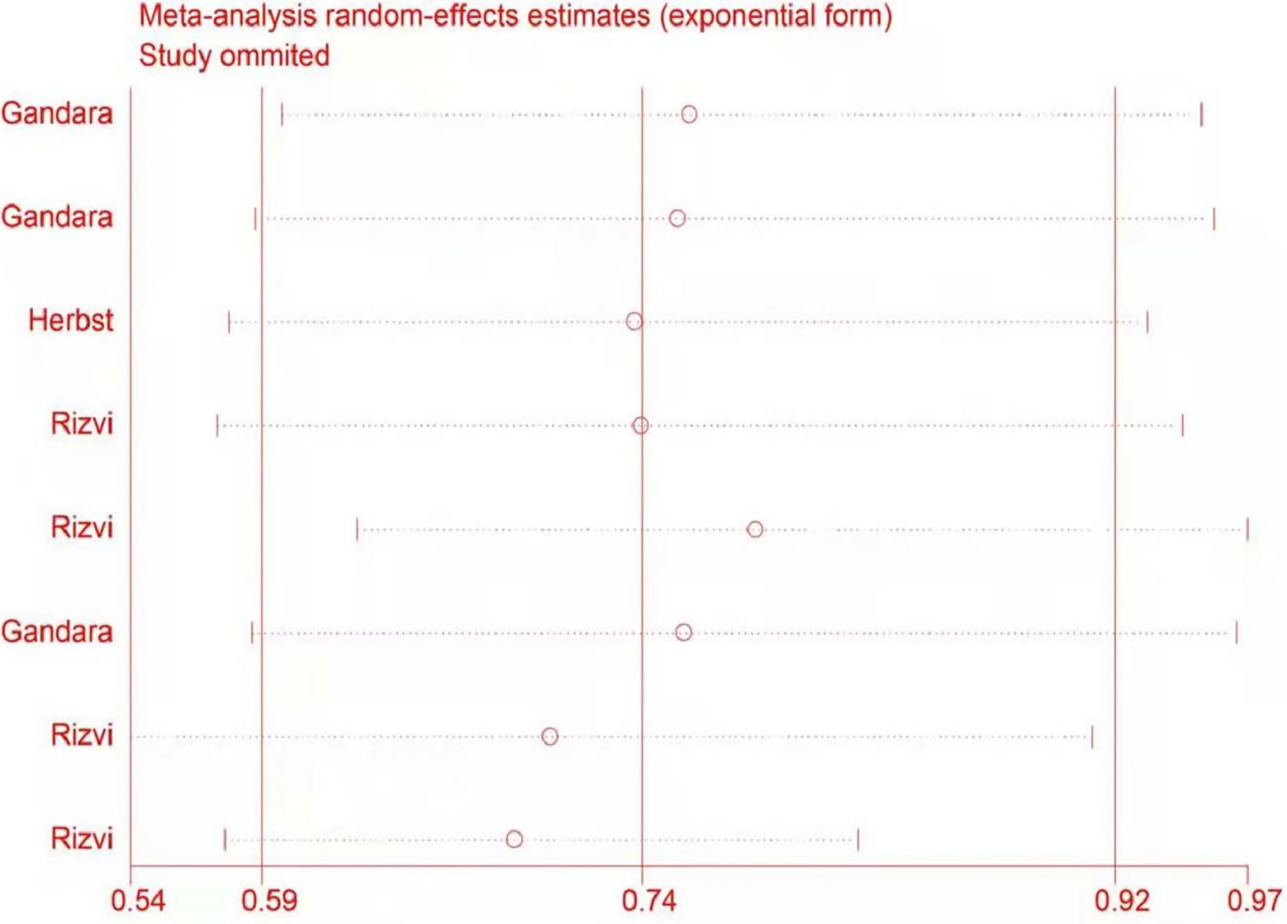
Sensitivity analysis of the potential impact of individual studies on outcomes.

The potential impact of individual studies on the pooled HR was assessed in a sensitivity analysis. There were no significant changes in any of the outcomes, indicating that the analysis was stable (**Figure 7**).

## Discussion

A decade ago, the term liquid biopsy was only used to describe the use of blood tests to provide the same diagnostic information as tissue biopsies (37). Liquid biopsies are more convenient, less invasive, and less expensive for patients than traditional tissue biopsies. In addition, their collection is less expensive. In theory, liquid biopsies could provide patients with more comprehensive information about tumor burden because the sample theoretically represents all tumor DNA that is present in the circulation, and they are superior to tissue biopsies, which are restricted to a single spatial anatomic site. The first liquid biopsy products based on EGFR gene mutations were approved by FDA in 2016 (38). So far, liquid biopsy has made a significant impact in the field of cancer treatment. However, recent reports on the predictive value of bTMB have been inconsistent. As a result, this meta-analysis focused on the clinical utility of bTMB as a predictive biomarker of immunotherapy response to stratify NSCLC patients treated with ICIs. In our study, we collected data from seven trials with a total of 2,610 patients to assess the predictive power of bTMB. A comparison of high bTMB versus low bTMB was performed in the ICIs cohorts. The pooled results revealed that there was no significant difference in long-term or short-term benefits. This finding is somewhat surprising, implying that bTMB does not appear to be effective in stratifying suitable patients for immunotherapy. Even if these findings contradict previous research, there is still evidence to support the situation (39). In our previous study, tissue-based TMB (tTMB) was thought to be more suitable for stratifying NSCLC patients treated with ICIs than bTMB (40). Patients with high bTMB had longer PFS in a small independent validation cohort in Chen’s (41) study, but the benefit was only a numerical trend with no statistical difference when compared to those with low bTMB. According to Chen’s research, this phenomenon may be caused by the fact that only 65% of tTMB and bTMB concordance exists and that less than 40% of identified variants are shared by both tumor samples and blood. We hypothesized that the deep reason stemmed from the inherent difficulties of circulating DNA analysis: low sensitivity. As is well known, tumors must shed DNA into the blood in order for ctDNA to be detected in the blood for optimal assay performance. The levels of ctDNA molecules in the blood, on the other hand, are typically much lower than levels of non-cancer-related DNA fragments in the blood. Although the majority of the tumor mutations were present in plasma, but at very low frequencies (1%), it was nearly impossible for the software to distinguish them from the background error noise (42). As a result, the low levels of mutant ctDNA fragments are obscured by a mass of background DNA, resulting in some mutations that can only be discovered in tumor tissues and not in blood samples, leading to false-negative results. Therefore, the low sensitivity of bTMB test may play an important role in the final negative results. According to Zhang’s (43) study, when using bTMB to define TMB-high patients, it demonstrated extremely high specificity (100%) but comparatively low sensitivity (43%), with false-negative predictions occurring more frequently in stage I–II patients and categorization precision being higher in stage III–IV patients than in other stage patients. Previous research discovered that accurate tumor information can be obtained only when the proportion of ctDNA is greater than or equal to 10% of cell-free DNA (cfDNA). With the exception of some advanced tumors that release large amounts of ctDNA, the vast majority of patients do not meet this requirement (44–46). As a result, we inferred that tumor stage may have an important impact on bTMB prediction results. In patients with a low stage, the predictive value of bTMB may be negative. Despite the fact that the majority of the subjects in this study were in stages III–IV, patients in stages I–II exist objectively, and the influence of tumor stage on the predictive value of bTMB required further validation. Intratumoral heterogeneity may also be an important factor causing the deviation. According to Gandara, intratumoral heterogeneity is the primary source of variation between bTMB and tTMB (25). Similarly, assay technologies and TMB assessment algorithms have a significant impact on the final results. BTMB was traditionally defined as the number of detected mutations across the sequenced ctDNA region (28). However, the bTMB calculation was not consistent. There are several types of mutations that can be used to count bTMB. This includes, but is not limited to, synonymous or nonsynonymous base substitutions, copy number variants, rearrangements, insertions, and deletions. On different gene panel platforms, bTMB calculation may select only one or two of these mutation types while ignoring the others. This causes a significant difference in bTMB test results, which may play an important impact on the final meta-analysis results. In 2018, the American Society of Clinical Oncology (ASCO) and the American College of Pathologists (CAP) formed an expert committee to review 1,338 published clinical ctDNA tests. After evaluating the ctDNA detection method from three perspectives: analytic validity, clinical validity, and clinical utility, they concluded that the current clinical efficacy of liquid biopsy techniques is very limited (47). In conclusion, blood-based TMB has the potential predictive efficacy of ICIs in NSCLC, but its utility is limited at this time, and more research is required.

This study compared ICIs to chemotherapy and performed a subgroup analysis based on different levels of bTMB. These findings suggested that in terms of overall survival, progression-free survival, and objective response rates, NSCLC patients with high bTMB may benefit more from ICIs therapy than chemotherapy. Patients with low bTMB, on the other hand, showed no significant differences between chemotherapy and ICIs therapy. Patients with low bTMB responded better to chemotherapy than immunotherapy, especially in terms of ORR. The results show that bTMB has a good screening function and can effectively screen patients for immunotherapy instead of chemotherapy. This discovery appears to contradict the preceding conclusion, but it is not surprising. Tumor mutation burden (TMB) has been repeatedly demonstrated as a predictive biomarker (48, 49), despite the fact that the above-mentioned findings do not support the predictive efficacy of bTMB. Based on the findings of KEYNOTE-158 (50), the FDA approved pembrolizumab on June 16, 2020, for the treatment of pediatric and adult patients with unresectable or metastatic tumor mutational burden-high (TMB-H) solid tumors (51). Our previous study (40) also confirmed that ICIs provided greater clinical benefit than chemotherapy in NSCLC patients with high TMB. Such findings simply suggest that the TMB assay derived from blood samples may reduce the predictive power of TMB for immunotherapy. The predictive efficacy of TMB is objective and real. Of course, we recognize that more detailed data on immunotherapy versus chemotherapy needs to be supplemented. More research is required. In conclusion, TMB as a predictive biomarker for ICIs therapy is feasible and reliable, and ICIs therapy may be a better option for NSCLC patients with high TMB.

In 2016, the open-label, randomized, phase III KEYNOTE-024 trial found that pembrolizumab was more effective than platinum-based chemotherapy in NSCLC patients with high levels of PD-L1 expression [tumor proportion score (TPS) of 50% or higher] (52). On the basis of the KEYNOTE-024 trial, the FDA approved pembrolizumab as first-line treatment for metastatic non-small cell lung cancer patients [PD-L1 expression: tumor proportion score (TPS) ≥50%] with no epidermal growth factor receptor (EGFR) or anaplastic lymphoma kinase (ALK) genomic tumor aberrations on October 24, 2016 (53). However, there have been so few open-label, randomized clinical trials that we have not been able to evaluate the efficacy of ICI as a first-line agent for NSCLC patients compared to standard treatment using bTMB as a biomarker due to a lack of available data. In the meantime, our meta-analysis has also some limitations. First and foremost, the heterogeneity of the studies is significant. Our findings were based on unadjusted analysis, and more precise results would be obtained by adjusting for other potential confounding factors such as gender, age, PD-L1 status, treatment, different panel-based sequencing, distinguished bTMB cut-off value, and so on. Despite using subgroup analysis to reduce heterogeneity, the results showed no improvement in meaning. Second, there were fewer trials included in this analysis, and the sample size varied among the included studies, raising the possibility of publication bias. Third, the majority of the studies included in the analysis are retrospective in nature, which may introduce selection bias and other uncontrolled variables into the assessment of bTMB and associated clinical outcomes. Fourth, some data in the meta-analysis overlapped. We ran a sensitivity analysis to eliminate the effect of each study on the results separately. Although the outcome of sensitivity analysis is stable, its impact on the final conclusion is objective and cannot be overlooked. Finally, the funnel plot and Egger linear regression test were not performed because, according to the Cochrane Collaboration Handbook (54), tests for publication bias were not validated if the number of studies involved was less than ten, so they were not performed. Given the foregoing, the finding of the study should be interpreted with caution, and additional validation trails are required.

### Conclusions

TMB is a feasible and reliable predictive biomarker for identifying NSCLC patients who are candidates for ICIs therapy. Although bTMB has the potential to predict, its role at this stage is limited. More prospective data is required. ICIs may be a better option than chemotherapy for NSCLC patients with high TMB.

## Data Availability Statement

The original datasets presented in the study are included in the article/supplementary material. Further inquiry can be directed to the corresponding author.

## Author Contributions

NZ helped to conceptualize and design the work. NZ and JZ helped to write the draft manuscript. NZ, XY, and XH helped with the literature search and data collection. XH, YM, and GW helped with data analysis and interpretation. NZ and JZ contributed to the supervision and critical revision of the manuscript. All authors listed have made a substantial, direct, and intellectual contribution to the work and approved it for publication.

## Conflict of Interest

The authors declare that the research was conducted in the absence of any commercial or financial relationships that could be construed as a potential conflict of interest.

## Publisher’s Note

All claims expressed in this article are solely those of the authors and do not necessarily represent those of their affiliated organizations, or those of the publisher, the editors and the reviewers. Any product that may be evaluated in this article, or claim that may be made by its manufacturer, is not guaranteed or endorsed by the publisher.
